# Repeat induced abortion and associated factors among reproductive age women who seek abortion services in Debre Berhan town health institutions, Central Ethiopia, 2019

**DOI:** 10.1186/s13104-019-4542-3

**Published:** 2019-08-13

**Authors:** Geremew Kindie Behulu, Endegena Abebe Fenta, Getie Lake Aynalem

**Affiliations:** 1 Department of Midwifery, Debre Berhan Health Sciences College, Debre Berhan, P.O. Box 37, Amhara region Ethiopia; 2 Department of Basic Health Science, Debre-Berhan Health Science College, Debre Berhan, Ethiopia; 3 School of Midwifery, College of Medicine and Health Science, University of Gondar, Gondar, Ethiopia

**Keywords:** Repeated abortion, Debre Berhan, Ethiopia

## Abstract

**Objective:**

To assess the magnitude and associated factors of repeat induced abortion among women aged from 15 to 49 who seek abortion care services in the health institutions of Debre Berhan town, Central Ethiopia, 2019.

**Results:**

This study shows that the prevalence of repeat induced abortion among 355 respondents was to be 20.3%. Those who reported as they had more than one partner in the last 12 preceding months, (AOR = 7.3, 95% CI 3.21, 16.46), Age of the first sexual intercourse less than 18 years (AOR = 6, 95% CI 2.54, 13.95) and Perceiving abortion procedure as it was not painful (AOR = 7.7, 95% CI 2.9, 20.6) were variables positively associated with the repeatedly induced abortion among women who sought abortion services.

## Introduction

Repeat-induced abortion defined as those reporting at least one previous induced abortion [1]. Abortion is a sensitive and controversial issue with religious, moral, cultural, and political scopes. It is also a public health issue in many parts of the world. More than one-fourth of the world’s people live in rural areas where the procedure is prohibited or allowed only to preserve the woman’s lifetime. However, irrespective of legal status, abortions still occur, and nearly half of them are performed by an unskilled practitioner or in less than sanitary conditions, or both [2].

The World Health Organization (WHO) estimates that worldwide 210 million women get pregnant each year and that nearly two-thirds of them, or roughly 130 million, deliver live babies. The remaining one-third of pregnancies end in miscarriage, stillbirth, or induced abortion [2]. Statistical reports show that around 13% of maternal deaths are contributed by unsafe abortion in the globe [3]. Induced abortion is frequently a consequence of inadequate contraception and the reasons not to use contraception originate from lack of correct information [4].

Repeat abortion, or getting more than one pregnancy termination, is bound in a vicious cycle with repeat unintended pregnancy [5]. Women who have had a recent abortion are more potential to discontinue contraceptive use during a 1-year follow up period, and both current and other previous abortion clients are more likely to have a repeat unintended pregnancy during that period [6]. The incidence of women looking for induced abortion and particularly those seeking a repeated induced abortion is an essential indicator of the frequency with which women have unintended pregnancies, and it can point to gaps in contraceptive services and effective contraceptive use [7].

Despite the high incidence of repeat abortions and their consequences, research on it is scarce in low and middle-income countries, particularly in Ethiopia. Abortion is currently legal in Ethiopia in cases of rape, incest, or fetal impairment. Also, a woman can legally terminate a pregnancy if her life or her child’s life is at risk, or if continuing the pregnancy or giving birth threatens her life. A woman may also terminate a pregnancy if she is unable to bring up the child, owing to her status as a minor or to a physical or mental infirmity since 2005 and the contraceptive coverage reached 36% in 2016 [8]. The abortion rate among childbearing age women was about 23 per 1000 women aged 15–44 in 2008 [9].

The magnitude of repeat abortion in Ethiopia is not known; this study seeks to help enlighten. The overall prevalence of unintended pregnancy in Ethiopia is about 42%. Among an annual 3.27 million estimated pregnancies, half a million ends up in abortion [10].

As far as our knowledge is concerned, there are no studies done on the magnitude and associated factors of repeat induced abortion in Amhara region, so the aim of this study was to determine the prevalence and associated factors of repeat induced abortion among the reproductive age group of women at the health institutions of Debre Berhan town, Central Ethiopia.

## Main text

### Methods

#### Study design and setting

An institutional based cross-sectional study design was conducted among the reproductive age group of women at the health institutions in Debre Berhan town. The town is found in North Shewa Zone, North West Ethiopia, Amhara region which is about 120 km from Addis Ababa (capital city of Ethiopia). Based on the 2007 national census carried on by the Central Statistical Agency of Ethiopia (CSA), this town has total residents of 65,231, of whom 31,668 are men and 33,563 women. The most of the peoples practiced Ethiopian Orthodox Christianity, with 94.12% reporting that as their religion, while 3.32% of the population said they were Muslim and 2.15% were Protestants [11]. There are two governmental and one private health institutions in the town.

#### Sample size and sampling procedure

Sample size calculation was done by using a single proportion population formula by considering the following assumptions: p = 0.16 from previous study [12], 95% CI, and 4% marginal error. Then, using the formula ${\text{n}}\, = \,{{\left( {\left( {{\text{Z}}\alpha / 2} \right) 2 {\text{ p }}\left( { 1\, - \,{\text{p}}} \right)} \right)} \mathord{\left/ {\vphantom {{\left( {\left( {{\text{Z}}\alpha / 2} \right) 2 {\text{ p }}\left( { 1\, - \,{\text{p}}} \right)} \right)} {{\text{W2}}.}}} \right. \kern-0pt} {{\text{W2}}.}}$

Where $${\text{Z}}\alpha / 2\, = \, 1. 9 6$$

$${\text{n}}\, = \,{{\left( {\left( { 1. 9 6} \right) 2 { }0. 1 6 { }\left( {0. 8 4} \right)} \right)} \mathord{\left/ {\vphantom {{\left( {\left( { 1. 9 6} \right) 2 { }0. 1 6 { }\left( {0. 8 4} \right)} \right)} {\left( {0.0 4} \right) 2\, = \, 3 2 2. 7}}} \right. \kern-0pt} {\left( {0.0 4} \right) 2\, = \, 3 2 2. 7}}$$

By adding a 10% non-response rate, the total sample size required was 355.

Systematic random sampling technique was used to get the participants. The 1 month preceded case flow of each health institution was determined. Accordingly, Debre Berhan Hospital (50 cases in a month), Marie stopes private clinic (115 cases in 1 month) and Debre Berhan health center (45 cases in a month) were recorded. The total population in the data collection period (from September 12/2018 to February 12/2019) was 1050. The K interval then became 3 and the first number to start collection was selected by lottery method which was 2. Then using the proportional sample size allocation according to the case flow, samples which were systematically selected from each institution were 85, 194 and 76 for Debre Berhan Hospital, Marie Stopes private clinic and Debre Berhan health center respectively.

#### Operational definition

##### Induced abortion

Intentional termination of pregnancy by any means or person other than spontaneous (excludes miscarriage) (WHO).

##### Repeat induced abortion

Women having more than one induced pregnancy termination defined in health care facility.

#### Data collection instrument and process

Data were collected using semi-structured, pretested, and face-to-face interview in a private room at the workplace in the exit time. The questionnaire was adapted from the literatures. The tool was prepared in English and then translated into the local language, Amharic, and finally returned to English for consistency checking. Six female diploma midwives and two female degree holder midwives as supervisors were involved in the data collection process.

#### Data quality control

Semi-structured data collection tool was utilized and clarity of the tool was tested before the final utilization. The pretest was conducted among 5% of the sample size in the other health institution which was out of the study area. A 1 day training was given for data collectors and supervisors regarding the objectives of the study, data collection method and significance of the study. During data collection each data collector was supervised for any difficulties and directions and necessary corrections were provided.

#### Data analysis

Data were coded and entered into a computer using Epi info version 7.2.0.1. It was then checked for completeness and transferred to SPSS version 23 for analysis. Univariate analysis including mean and different frequencies were done. Crudely associated variables were identified by bivariate logistic regression model and these variables were fitted to multiple logistic regression. Then association between dependent and the explanatory variable was assessed using adjusted Odds Ratio (AOR), 95% CI and *p* value of ≤ 0.05 were considered statistically significant.

### Results

#### Socio-demographic characteristics

From the selected 355 participants, a total of 345 completed the questionnaire whereas ten refused to participate in the study, giving a response rate of 97.18%. The median age of the study participants was 27 with the interquartile range of 6. The maximum age was 40 years, and the minimum age was 17 years. Three hundred eight (89.3%) were urban resident. One hundred eighty-five (53.6%) were college diploma and above as to their educational status. The Majority of the respondents, 295 (85.5%) were followers of Orthodox Christianity. Two hundred sixty-four (76.5%) respondents were single (Table 1).

**Table 1:** Socio-demographic characteristics of the participants who sought abortion services in health institution of Debre Berhan town, from Sep. 12/2018 to Feb. 12/2019 (n = 345)

Variables	Frequency	Percent (%)
Age		
15–20	49	14.2
21–25	78	22.6
26–30	147	42.6
31–35	41	11.9
36–40	30	8.7
Place of residence		
Urban	308	89.3
Rural	37	10.7
Educational status		
College diploma and above	185	53.6
Elementary	50	14.5
Secondary	110	31.9
Religion		
Muslim	25	7.2
Orthodox	295	85.5
Protestant	25	7.2
Ethnicity		
Amhara	265	76.8
Oromo	59	17.1
Tigre	21	6.1
Marital status		
Divorced	27	7.8
Married	54	15.7
Single	264	76.5
Occupation		
Daily laborer	25	7.2
Farmer	25	7.2
Gov. employee	46	13.3
House-made	25	7.2
Housewife	33	9.6
Merchant	25	7.2
Private employee	36	10.4
Sex worker	25	7.2
Student	105	30.4
Own income		
No	164	47.5
Yes	181	52.5

#### Reproductive health characteristics

From the total participants, three-hundred thirty-nine (98.3%) responded that their last pregnancy was not wanted and seventy (20.3%) of respondents reported that they had repeat induced abortion. Eighty-five of the participants (24.6%) age of first sexual intercourse was less than eighteen years. The main reasons to have repeat induced abortion mentioned by the participants were: Economic problem (41%), Being a student (27%), Raped (16%) and separated from the husband (16%). Ninety-six (27.8%) of respondents had more than one sexual partner. Two hundred ninety-one (84.3%) respondents were ever not used family planning and three hundred nineteen (92.5%) was planned to use family planning (Table 2).

**Table 2:** Reproductive health characteristics of reproductive age women who sought abortion services in Debre Berhan town health institution, from Sep. 12/2018 to Feb. 12/2019 (n = 345)

Variables	Frequency	Percent (%)
Last pregnancy wanted		
No	339	98.3
Yes	6	1.7
Had repeat induced abortion		
No	275	79.7
Yes	70	20.3
Method used		
Medication	141	40.9
MVA	204	59.1
Place of service		
Hospital	82	23.8
Private clinic	188	54.5
Public health centers	75	21.7
Help to get the service		
Boyfriend	30	8.7
Friend	224	64.9
Husband	40	11.6
Nobody	26	7.5
Sister	25	7.2
Age of first sexual intercourse		
< 18	85	24.6
≥ 18	260	75.4
Number of sexual partner		
One	249	72.2
Two and above	96	27.8
Children for the future		
No	50	14.5
Yes	295	85.5
Procedure was painful		
No	51	14.8
Yes	294	85.2
Ever use family planning		
No	291	84.3
Yes	54	15.7
Ever use emergency contraceptive		
No	320	92.8
Yes	25	7.2
Plan to use family planning		
No	26	7.5
Yes	319	92.5

#### Associated factors of repeat induced abortion

Crudely associated variables were: age, Place of residence, Marital status, income, Number of sexual partners, Age of first sexual intercourse, Occupation, Ever use family planning and perceiving Procedure was painful.

Independently and positively associated variables in adjusted analysis were: Having more than one sexual partner in preceding 12 months, conducting sexual intercourse less than eighteen years and perceiving the previous abortion procedure as it was not painful (Table 3).

**Table 3:** Both bivariate and multivariate analysis of factors

Variables	Repeat induced abortion		COR (95% CI)	AOR (95% CI)
	Yes	No		
Age				
15–20	3	46	1	
21–25	15	63	3.65 (0.99, 13.35)	
26–30	26	121	3.29 (0.95, 11.41)	
31–35	19	22	13.2 (3.54, 49.54)***	
36–40	7	23	4.66 (1.10, 19.73)*	
Place of residence				
Urban	68	240	4.95 (1.16, 21.14)*	
Rural	2	35	1	
Marital status				
Divorced	12	15	3.34 (1.47, 7.57)**	
Married	7	47	0.62 (0.26, 1.45)	
Single	51	213	1	
Own income				
No	48	116	2.99 (1.71, 5.22)***	
Yes	22	159	1	
Number of sexual partner				
One	29	220	1	1
Two and above	41	55	5.65 (3.23, 9.89)***	7.72 (2.90, 20.58)***
Age of first sexual intercourse				
≥ 18	32	228	1	1
< 18	38	47	5.76 (3.27, 10.14)***	5.96 (2.54, 13.95)***
Occupation				
Daily laborer	1	24	0.18 (0.02, 1.48)	
Farmer	12	13	4.17 (1.65, 10.57)	
Gov. employee	11	35	1.42 (0.61, 3.29)	
House-made	4	21	0.86 (0.265, 2.80)	
Housewife	2	31	0.29 (0.06, 1.32)	
Merchant	8	17	2.13 (0.80, 5.65)	
Private employee	3	33	0.41 (0.11, 1.48)	
Sex worker	10	15	3.01 (1.17, 7.73)*	
Student	19	86	1	
Ever use family planning				
No	66	225	3.66 (1.27, 10.52)*	
Yes	4	50	1	
Procedure was painful				
No	19	32	2.82 (1.48, 5.38)**	7.27 (3.21, 16.46)***
Yes	51	243	1	1

* p-value < 0.05, **p-value < 0.01 and ***p-value < 0.001

### Discussion

This institutional based cross sectional study has attempted to assess the repeated induced abortion and associated factors among reproductive age women who sought abortion service in Debre Berhan town, North Shewa, Amhara region, Central Ethiopia, 2019. The study revealed that the prevalence of repeated induced abortion was 20.3% with 95% CI of (16.4, 24.3). This finding was in line with the study from Kenya, 16% [12].

On the other hand, this study’s finding was lower than the study from Italy, 60.6% [1]. This difference could be explained by the variance in a background of the study participants, variation in the study area, the cultural dissimilarity between countries, and high family planning coverage in developed countries.

This study revealed those who have more than one sexual partner in the preceding 12 months were seven times more likely to engage in repeat induced abortion when compared to those who have a single sexual partner (AOR = 7.27, 95% CI 3.21, 16.46). It was consistent with studies from Northern Ethiopia [13] and Cambodia [14]. A possible explanation for this tendency is the increased probability of condom failure, corresponding to the increased number of sexual intercourse. The Government should continue to encourage women to define and trim down their number of sexual partners, both as a means to reduce HIV and STI transmission.

Besides, sexual debut before 18 years was a predictor variable (AOR = 5.96, 95% CI 2.54, 13.95). It was similar to the study from Northern Ethiopia [13]. This might due to those who experience sexual intercourse exposed to a different sexual partner and fail to use a contraceptive. The Government should work to help adolescents delay sexual debut and encouraging family planning, including empowering communities and especially women, to freely discuss sexuality with young girls at home.

The other positive predictor of repeat induced abortion was that perceiving abortion procedure is not painful were eight times more likely to be exposed to repeated induced abortion (AOR = 7.72, 95% CI 2.90, 20.58). It was consistent with the study from Northern Ethiopia [13]. This might due to considering the procedure not painful as well as abortion as the family planning method.

## Limitations

May be affected by cross-sectional study design draw backs and comparing its findings with the community based studies may be difficult since it is institutional based.

## Data Availability

The datasets used and/or analyzed during the current study are available from the corresponding author on reasonable request.

## Abbreviations

AOR: adjusted odds ratio
CSA: Central Statistical Agency of Ethiopia
FMOH: Federal ministry of health
MVA: manual vacuum aspiration
WHO: World Health Organization
